# Epidemiology of cancer in older adults: a systematic review of age-related differences in solid malignancies treatment

**DOI:** 10.1007/s11912-025-01638-6

**Published:** 2025-02-15

**Authors:** Esther Bastiaannet, Sophie Pilleron

**Affiliations:** 1 Epidemiology, Biostatistics and Prevention Institute, University of Zurich, Hirschengraben 84, CH-8001 Zurich, Switzerland; 2 Ageing, Cancer, and Disparities Research Unit, Department of Precision Health, Luxembourg Institute of Health, 1A-B, Rue Thomas Edison, 1445 Strassen, Luxembourg

**Keywords:** Epidemiology, Aging, Therapy, Outcomes

## Abstract

**Purpose of review:**

We examined the latest epidemiological research on age-related differences in cancer treatment and selected outcomes, among patients with cancer aged 60 and above in comparison to younger patients.

**Recent findings:**

Colorectal, pancreatic and lung cancers were studied most often. Most studies were conducted in Europe or the United States of America (USA) within single centers. For unselected patients, older patients receive less treatment, and their survival, regardless of the metric used (cancer-specific survival or overall survival), was poorer than that of middle-aged patients. Age-related differences in treatment and outcomes were more pronounced in patients aged over 80 years. However, among patients selected for treatment, complications, adverse events rates and survival probabilities were comparable between older and younger patients. Treatment differences, especially the omission of therapy, were often smaller for good prognosis cancer types.

**Summary:**

The likelihood of receiving treatment decreased as age increases, regardless of the cancer types, treatment, countries and setting. More research on treatment in older patients with cancer, especially the frailest and the oldest, is urgently needed as there is still a lack of data to tailor treatment.

## Introduction

Due to the ageing of the global population, cancer burden in older adults is increasing. Worldwide, the number of people aged 65 years or older is projected to more than double [1], with an even faster growth in people over the age of 80 years [2, 3]. Most older people are fit, however, others might be frail or chronically ill as a result of the physiological changes in organ systems, more prevalent diseases, and other changes associated with ageing [4]. As adults age in different ways and with different trajectories, the older population is very heterogeneous in terms of health and functional status [5]. The presence of comorbidities, poorer health status and advanced cancer stage at diagnosis, as well as patient preferences, are usually mentioned to deviate from guideline adherent treatment for selected older patients.

Epidemiological observational studies play a crucial role in the (post market approval) evaluation of treatment and outcomes as they provide real-world evidence for all patients, identify complications and adverse events and study long-term safety [6]. In particular, observational studies can be useful studying large heterogeneous patient populations and highlight disparities across age groups, thereby guiding research and interventions. Over the past years, literature on cancer treatment in older patients has grown; there are, however, conflicting results on the treatment differences between younger or middle-aged and older patients, differences with respect to changes in treatment over time, and not all tumor types have been described. In addition, the settings (single center reports or larger population-based studies), countries and age cut-off are mostly scattered. As the number of publications in the geriatric oncology field has increased in recent years, it is crucial to provide an overview of the recent literature on treatment in older adults. Therefore, this systematic review aims to synthesize the latest research on the age-related differences in cancer treatment, and selected outcomes, among patients with cancer aged 60 and above in comparison to younger patients.

## Methods

### Search Strategy and Article Selection

The literature review protocol was pre-registered in PROSPERO (CRD42023450654); results are described in two papers, one focusing on prevalence, incidence, and mortality in general and more specifically on age-related survival differences and associated factors [7]; and the present one focusing on age-related differences in treatment strategies and associated outcomes. We searched papers published from January 1st, 2019 until August 3, 2023 in Embase and MEDLINE. The search strategy was first set up in MEDLINE and then adapted for Embase using search terms corresponding to “older adults”, “cancer”, and “therapy”. Older adults were defined as 60 years or older following the definition of the United Nations.

**Fig. 1:**
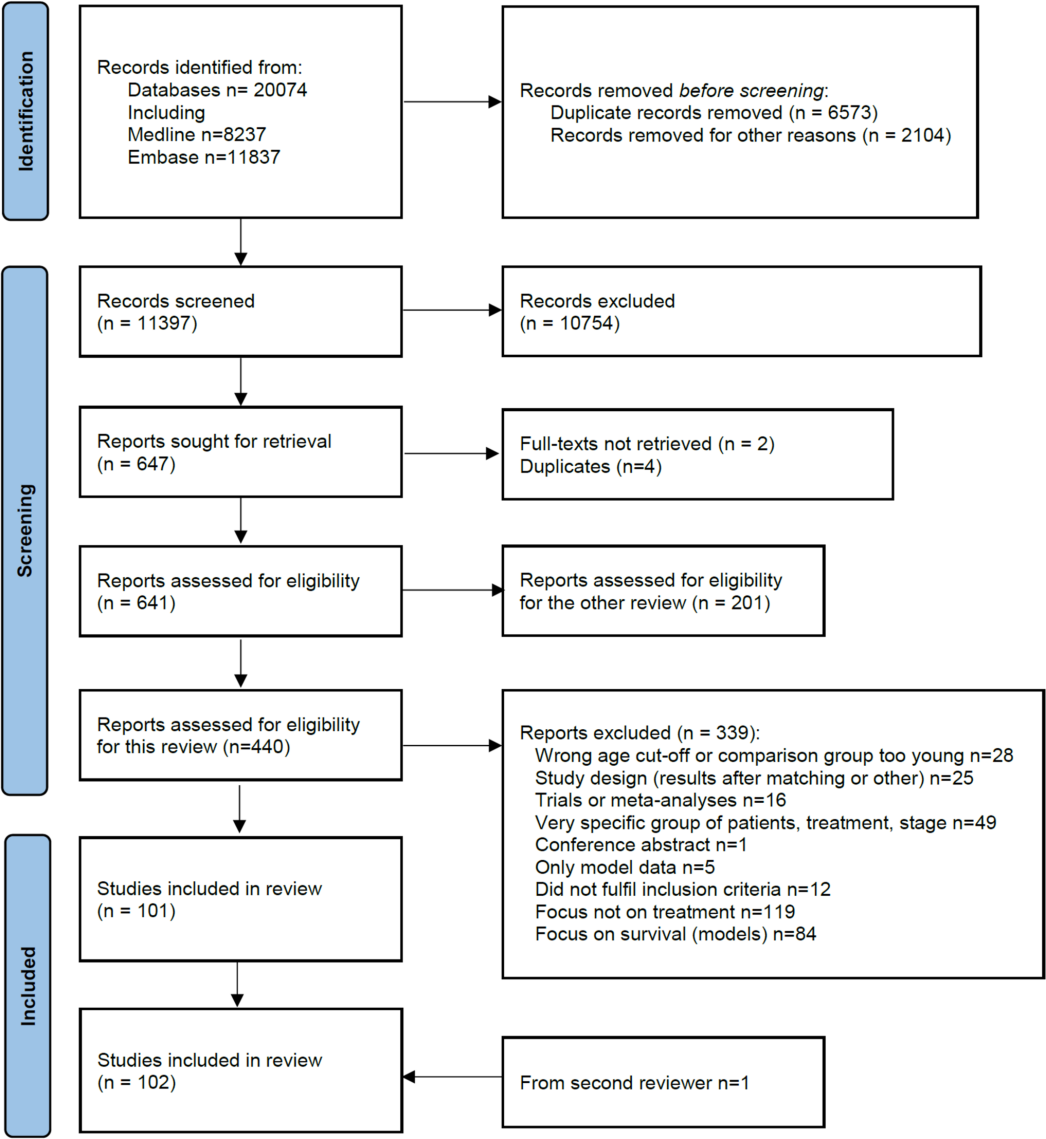
Flow diagram of the review

Figure 1 shows the selection of articles. Duplicate records were removed before the screening. Two independent reviewers screened titles and abstracts with the following inclusion criteria for the present review: 1) the paper was primarily focused on treatment differences; 2) between young and older patients (60 years or older, any cut-off was acceptable) with cancer; and 3) published in English. Exclusion criteria were: 1) no age group comparison in the paper or only two extreme age groups compared (e.g. 80 + versus 40 and younger), 2) focus only on survival outcomes, 3) inclusion of a small subset of patients with a specific stage (e.g. stage Ia) or studies that assessed a new surgical technique or a new or very selected (non-standard) systemic treatment. Conference abstracts, reviews of any kind, editorials, or letters to editors were also excluded. There was no restriction on the study outcomes and geography. Any disagreements were resolved by referral to a third reviewer. Next, the full text of all remaining papers was obtained.

### Data Extraction

First, general study characteristics as first author, year of publication, cancer site, country, study years, setting and the number of patients in the study were extracted from the articles. With respect to the setting, population-based studies had to include all consecutive patients from a registry, single center from a single hospital or cancer center. Studies were sorted on the cancer localization. Next, the following data with respect to studies reporting treatment differences in general were extracted: age categories as used in the article, treatment percentages for the younger and older age groups and in some papers for a middle age group or an old age group defined as 80 years or older, p-values for the difference and a summary of the differences in outcomes. Studies were sorted by the age cut-off and cancer type (based on low or high survival). Finally, the following data was extracted from studies that described age-related differences in populations receiving a specific treatment: cancer site, age cut-off, treatment and outcomes, percentages in the age groups and p-value; for articles that described surgical treatment we extracted 30-day (or 90-day) mortality, complications or adverse events (when combined with systemic treatment), and survival or recurrence data. When an article reported several specified adverse events, only the combined percentages or the most frequent adverse event was extracted. For studies reporting about age-related differences in systemic treatment, we additionally extracted data with respect to adverse events or toxicity, information on the dose or number of cycles, and time to progression where applicable. Zotero V.6.0.36 was used to manage references and Rayyan [8] for the title, abstract and full-text screening.

## Results

Overall, we selected 440 papers for full-text review and excluded 339 papers, subsequently, 101 papers were included, and 1 study that fulfilled the inclusion criteria, however not present in the full-text review selection, was added by SP [7], resulting in the inclusion of 102 articles. Table 1 describes the characteristics of the included studies, sorted by tumor type. Fifteen studies focused on tumors specifically in females, namely breast [9–12], cervical [13–16], ovarian [17–20], endometrial [21, 22] or vulvar [23] cancers. A large proportion of the studies assessed age-related differences in treatment for rectal (n = 10) [24–33], colon (n = 6) [34–39], or colorectal cancers (n = 7) [40–46]. Gastric cancer was studied in 5 studies [47–51] and esophageal cancer in 7 studies [52–58]. A large number of studies focused on pancreatic cancer (n = 12) [59–70], and lung cancer (n = 9) [71–79] including Small Cell Lung Cancer (SCLC) [78] and Non-Small Cell Lung Cancer (NSCLC) [74–77, 79]. Seven studies assessed age-related differences in treatment and outcomes in the metastatic setting [80–86]. The largest number of studies used data from Europe (36%), 24 studies from the USA (23%), and 15 from Japan (15%). Most studies were from large single centers (56%), 29 were population-based (28%), and the remaining were multicenter studies.

**Table 1:** Characteristics of the included studies on age-related differences in treatment by cancer site

First author, year	Cancer site	Country	Study year*	Setting**	Nr patients^#^
Tan, 2021[12]	Breast	Malaysia	2010–2014	Population-based	2166
Drapalik, 2022[9]	Breast	USA	2005–2017	Population-based	172272
Jauhari, 2019[10]	Breast	UK	2014–2016	Population-based	12716
Buonomo, 2023[11]	Breast	Italy	2016–2022	Single center	231
Hou, 2020[13]	Cervical	Taiwan	2007–2016	Single center	123
Xie, 2020[14]	Cervical	USA	2004–2015	Population-based	36816
Barben, 2022[15]	Cervical	France	2005–2015	Population-based	292
Neumeyer, 2023[16]	Cervical	Germany	2004–2013	Population-based	14528
Joueidi, 2020[17]	Ovarian	France	2000–2016	Multicenter	979
Pinelli, 2021[18]	Ovarian	UK	2016–2018	Single center	114
Zambrano-Vera, 2021[19]	Ovarian	USA	1999–2018	Single center	148
Van Walree, 2019[20]	Ovarian	Netherlands	2010–2015	Single center	128
Hotton, 2020[21]	Endometrium	France	2007–2016	Single center	148
Luzarraga-Aznar, 2022[22]	Endometrium	Spain	2010–2019	Single center	281
Hellman, 2020[23]	Vulva	Sweden	2012–2016	Population-based	657
Peltrini, 2021[24]	Rectal	Italy	2011–2020	Multicenter	287
Sonal, 2023[25]	Rectal	USA	2004–2018	Population-based	328
Mourad, 2021[26]	Rectal	Australia	2006–2018	Single center	699
Inanc, 2022[27]	Rectal	Turkey	2011–2018	Single center	175
De Nes, 2022[28]	Rectal	Netherlands	2008–2016	Population-based	6524
Birch, 2019[29]	Rectal	UK	2009–2014	Population-based	52922
Kang, 2021[30]	Rectal	Australia	2006–2015	Multicenter	736
Høydahl, 2022[31]	Rectal	Norway	1980–2016	Single center	666
Liu, 2020[32]	Rectal	China	2010–2018	Single center	414
Chesney, 2020[33]	Sigmoid & rectal	Canada	2002–2018	Single center	792
Hayes, 2019[34]	Colon	UK	1999–2010	Population-based	31910
Liu, 2023[35]	Colon	China	2004–2017	Single center	416
Mazzola, 2023[36]	Colon	Italy	2015–2018	Single center	130
Oytun, 2022[37]	Colon	Turkey	2010–2015	Single center	465
Shafiei, 2020[38]	Colon	Australia	2000–2010	Single center	1135
Hagerty, 2022[39]	Colon	USA	2010–2016	Single center + registry	14966
Ogata, 2022[40]	Colorectal	Japan	2013–2017	Single center	346
Cheng, 2022[41]	Colorectal	China	2011–2020	Single center	2084
Shiraishi, 2023[42]	Colorectal	Japan	2013–2021	Single center	215
Kryzauskas, 2021[43]	Colorectal	Lithuania	2014–2018	Single center	900
Cross, 2021[44]	Colorectal	Australia	2007–2018	Multicenter	20463
Okamoto, 2022[45]	Colorectal	Japan	2010–2021	Single center	138
Sarasqueta, 2019[46]	Colorectal	Spain	2010–2012	Single center	1157
Miller, 2022[87]	Anus	USA	2004–2015	Population-based	26796
Lee, 2022[105]	Gastrointestinal	Korea	2018–2020	Single center	477
Xu, 2021[47]	Gastric	China	2009–2014	Single center	306
Otowa, 2019[48]	Gastric	Japan	2014–2017	Single center	195
Keywani, 2023[49]	Gastric	Netherlands	2015–2019	Population-based	1995
Komori, 2020[50]	Gastric	Japan	2000–2012	Single center	411
Esaki, 2019[51]	Gastric	Japan	2000–2011	Multicenter	1969
Laurent, 2022[52]	Esophageal	Belgium	2006–2015	Single center	248
Kanda, 2019[53]	Esophageal	Japan	2005–2017	Single center	150
Cooper, 2021[54]	Esophageal	USA	2016–2020	Single center	201
Suzuki, 2022[55]	Esophageal	Japan	2010–2020	Single center	174
Bakhos, 2019[56]	Esophageal	USA	2004–2014	Population-based	107921
Baranov, 2022[57]	Esophageal	Netherlands	2011–2019	Audit	3775
Klevebro, 2019[58]	Esophageal, Gastric	Sweden	2007–2017	Single center	548
Vithayathil, 2022[92]	Hepatocellular	International	2020–2021	Single center	191
Marta, 2021[106]	Hepatocellular	Brazil	2007–2017	Single center	238
Shimada, 2020[107]	Hepatocellular	Japan	2000–2017	Single center	796
Inoue, 2019[108]	Hepatocellular	Japan	2001–2016	Single center	530
Liu, 2021[109]	Liver	Australia	2001–2017	Single center	357
Van Dongen, 2022[59]	Pancreas	Netherlands	2015–2018	Population-based	10298
Ramanathan, 2019[60]	Pancreas	USA	2014–2015	Population-based	1626
Gruppo, 2020[61]	Pancreas	Italy	2012–2017	Single center	124
Hackner, 2022[62]	Pancreas	Germany	2000–2018	Single center	213
Li, 2020[63]	Pancreas	USA	2004–2015	Population-based	140678
Elias, 2022[64]	Pancreas	USA	2015–2020	Population-based	5973
Henry, 2022[65]	Pancreas	Netherlands	2014–2016	Multicenter	836
Brada, 2021[66]	Pancreas	Netherlands	2015–2017	Multicenter	422
Sawyer, 2021[67]	Pancreas	USA	2011–2019	Single center	225
Izumo, 2021[68]	Pancreas	Japan	2000–2018	Single center	579
Oba, 2021[69]	Pancreas	USA	2011–2019	Single center	246
Malik, 2020[70]	Pancreas	UK	2005–2014	Single center	222
Sahli, 2021[110]	Thyroid	USA	2004–2015	Population-based	1457
Matrone, 2020[111]	Thyroid	Italy	2000–2018	Single center	432
Walter, 2019[71]	Lung	Germany	2009	Population-based	17478
Pham, 2021[72]	Lung	Australia	2011–2017	Population-based	3481
De León, 2021[73]	Lung	USA	2016–2019	Single center	673
Cao,2019[112]	LCNEC	USA / China	2004–2013	Population-based	1619
Grosjean, 2021[74]	NSCLC	Canada	2010–2019	Single center	327
Galli, 2019[79]	NSCLC	Italy	2013–2019	Single center	290
Zaborowska-Szmit, 2021[75]	NSCLC	Poland	2010–2014	Single center	196
Okishio, 2020[76]	NSCLC	Japan	2016–2016	Multicenter	901
Pilleron, 2023[77]	NSCLC	UK	2014–2017	Population-based	20716
Takeda, 2023[78]	SCLC	Japan	2019–2022	Single center	155
Linton, 2019[113]	MPM	Australia	2002–2009	Registry	1121
Pan, 2022[114]	MPM	USA	1975–2016	Population-based	1492
Lemiński, 2022[89]	Bladder	Poland	2003–2021	Single center	568
Sirithanaphol, 2019[115]	RCC	Thailand	2007–2017	Single center	101
Nemoto, 2022[91]	RCC	Japan	2013–2020	Multicenter	149
Bryant, 2022[116]	Prostate	USA	2000–2015	Population-based	12784
Thakur, 2020[117]	Meningioma	USA	2008–2019	Single center	291
Perla, 2021[118]	Infratentorial	USA	2012–2018	Population-based	2212
Gingrich, 2019[119]	STS	USA	2004–2012	Population-based	33859
Guertin, 2023[88]	STS	USA	2000–2015	Population-based	24666
Kotchetkov, 2023[93]	Lymphoma	Canada	2013–2022	Single center	201
Johns, 2021[120]	Mixed	USA	2011–2018	Population-based	673
Nia, 2020[121]	Mixed	USA	2008–2016	Multicenter	30,183
Storm, 2022[122]	Mixed	Netherlands	2016–2019	Single center	217
**Metastatic setting**					
Prager, 2021[80]	Pancreas	Austria	2015–2019	Multicenter	317
Koga, 2022[81]	Pancreas	Japan	2013–2017	Multicenter	153
Niedersüß-Beke, 2021[82]	Colorectal	Austria	2005–2020	Single center	1105
Potthoff, 2020[83]	Breast	Germany	2012–2015	Multicenter	407
Araujo, 2021[84]	RCC	International	2009–2019	Multicenter	1427
Lemelin, 2020[85]	Neuroendocrine	France	1990–2017	Single center	866
Liao, 2022[86]	Gastric	Taiwan	2009–2019	Single center	428

^*^Only study year extracted, irrespective of the months, **Population-based: consecutive patients from a registry, single center from a single hospital or cancer center, ^#^studies including a relatively small number (< 100 patients in total) of patients were excluded, MPM = Malignant pleural mesothelioma, LCNEC = large cell neuroendocrine carcinoma, RCC = Renal cell carcinoma, STS = Soft tissue sarcoma

### Age-Related Treatment Differences for Unselected Patients (Table 2, part A)

**Table 2:** Part A: Age-related differences in treatment for unselected patients, by age cut-off and tumour type sorted by population 5-year survival (high > 50% and low ≤ 50% (International Agency for Research on Cancer, World Health Organization)). Part B: Age-related differences in treatment and outcomes for specific treatment-selected patients

A. Age-related differences in treatment for unselected patients, by age cut-off and tumour type sorted by 5-year survival (high > 50% and low ≤ 50%)										
Author, year	Cancer site	Age		Treatment	Young (%)	Middle (%)	Older 60 + , 65 + , 70 + , 75 + (%)	Old 80 + (%)	p-value	Outcome
Cut-off 60 years										
Tan, 2021[12]	Breast (high)	< 40, 40–59, ≥ 60		No treatment Surgery only Surgery + oncologic Oncologic therapy	25.7 33.0 23.9 17.4	24.6 34.1 27.7 13.6	26.7 39.9 21.1 12.4			CSS and OS worse
Hellman, 2020[23]	Vulvar (high)	20–59, 60–69, 70–79, ≥ 80		Surgery Definitive RT No treatment	86 11 1	91 8 < 1	76 19 5	76 12 11	0.014 0.80 < 0.001	5-year OS and RS worse
Hayes, 2019[34]	Colon (high)	< 60,60–69,70–79, ≥ 80		Surgery CT surgical pts CT non-surgical pts	84.1 59.1 62.2	84.0 46.1 42.3	79.4 26.8 22.8	61.1 4.8 3.4	< 0.001 < 0.001 < 0.001	
Van Dongen, 2022[59]	Pancreas (low)	< 60, ≥ 60		Resection Neoadjuvant CRT Adj. chemotherapy Palliative CRT No treatment	22 18 71 43 33		14 11 53 18 67			Median OS worse
Pham, 2021[72]	Lung (low)	< 60,60–69,70–79, ≥ 80		Radiotherapy Surgery Chemotherapy Immunotherapy Any treatment	54.7 26.0 71.9 20.0 93.2	47.7 25.8 61.6 14.4 89.0	48.9 24.7 52.6 10.5 86.6	47.8 14.9 26.5 9.5 71.8	< 0.001	Median survival lower
Cut-off 65 years										
Sahlia, 2021[110]	Thyroid (high)	18–64, 65–79, ≥ 80		No surgery Lobectomy Total thyroidectomy	3.9 5.5 90.7		4.9 7.3 87.8	11.5 10.3 78.2	0.006	CSM worse
Xie, 2020[14]	Cervical (high)	< 65, ≥ 65		Surgery Radiotherapy Chemotherapy	62.30 52.93 47.25		36.52 65.30 47.02		< 0.001 < 0.001 0.741	
Neumeyer, 2023[16]	Cervical (high)	< 65, ≥ 65		Any treatment Surgery (stages) Chemotherapy Radiotherapy	44 18–46 8–35 12–45		40 15–46 5–14 18–43			5-year RS worse
Miller, 2022[87]	Anal (high)	< 65, ≥ 65		Concurrent CRT Local therapy Palliative therapy No treatment	65.0 21.4 11.2 2.4		54.5 25.1 16.1 4.3		< 0.001	
Mourad, 2021[26]	Rectal (high)	≤ 65, 66–79, ≥ 80		No surgery No treatment (non-surg pts)	10 23		16 33	29 47	< 0.001 0.005	
Høydahl, 2022[31]	Rectal (high)	< 65, 65–79, ≥ 80		Resection curative Res. noncurative Bypass or stoma	66 12 5	72 8 10		47 5 26	< 0.001	Death < 90d higher, OS & RS worse
Cao, 2019[112]	LCNEC (low)	< 65, ≥ 65		No Surgery Chemotherapy Radiotherapy	57.06 60.08 43.62		61.57 43.93 33.26		< 0.001 < 0.001 < 0.001	5-yr CSS worse
Walter, 2019[71]	Lung (low)	≤ 65, 65–74, 75–84, ≥ 85		No treatment Radiotherapy Tumor resection	4.4 23.7 34.7	7.8 21.9 35.4	20.2 21.4 27.8	54.7 17.1 15.3	< 0.0001 0.004 < 0.0001	
Pan, 2022[114]	MPM (low)	< 65, ≥ 65		Surgery Radiotherapy Chemotherapy	47.88 3.75 59.7		33.38 2.26 44.21		< 0.001 0.1 < 0.001	5-year CSS worse
Brada, 2021[66]	Pancreas, locally advanced (low)	< 65, 65–74, ≥ 75		Best supp care Folfirinox	12 78	18 63	46 14		< 0.01	Median OS worse
Cut-off 70 years										
Jauhari, 2019[10]	Breast, DCIS (high)	< 70, ≥ 70		Surgery Post-BCS RT	93.7 58.4		80.5 43.1			
Drapalik, 2022[9]	Breast, TN (high)	≤ 40, 41–70, > 70		No Chemotherapy Neoadjuvant CT Bilateral mastectomy No axillary surgery No radiation	11 44.1 37 12 50	19 30.1 17 12 42	57 20.5 4 21 55		< 0.001	
Barben, 2022[15]	Cervical (high)	< 70, ≥ 70		No treatment Surgery Lymphadenectomy Radiotherapy Chemotherapy	4.4 81.7 52.9 49.6 48.2		18.7 37.5 17.0 67.2 39.1		< 0.001 < 0.001 < 0.001 0.012 0.19	5-year survival worse
Kang, 2021[30]	Rectal, localized (high)	< 70, ≥ 70		Non-operative St III surgery alone Neoadjuvant stage III Adjuvant stage III Discussed MDT	2.0 4.3 54.3 79.1 59.5		7.4 25.2 36.8 47.1 50.2		0.0014 < 0.001 0.007 < 0.001 0.01	5-yr OS and CSS worse
De Nes, 2022[28]	Rectal, locally advanced (high)	< 70, ≥ 70		No treatment Resection only Neoadj + resection Adjuvant treatment	1.6 4.7 84.4 9.8		10.2 8.3 62.6 4.6		< 0.001	30-d and 90-d higher
Birch, 2019[29]	Rectal (high)	< 70, 70–79, ≥ 80		No surgery Major resection No radiotherapy Stoma creation	16.0 66.5 46.4 73.9		21.2 60.6 55.4 71.8	47.5 31.7 69.5 65.6		Deaths < 30d higher
Linton, 2019[113]	MPM (low)	< 70, 70–79, ≥ 80		Chemotherapy Surgery Adjuvant RT	62.6 14.4 8.6		35 1.1 1.1	8.1 0.6 0	< 0.001 < 0.001 < 0.001	Survival in months lower
Cut-off 75 years										
Gingrinch, 2019[119]	STS Extremities (high)	18–74, ≥ 74		No surgery Radiation Chemotherapy Palliative treatment	6.2 48.7 29.1 0.8		11.7 46.6 14.8 1.9		< 0.0001 0.002 < 0.0001 < 0.0001	90d mortality higher
Van Walree, 2019[20]	Ovarian (low)	< 75, ≥ 75		Discussed MDT Chemotherapy Supportive care only	87 89 7		64 52 38		0.002 < 0.001 < 0.001	
Cut-off 80 years										
Guertin, 2023[88]	STS (high)	< 80, ≥ 80		Surgery Radiotherapy Chemotherapy	86.2 41.5 20.7			74.0 39.1 3.5	< 0.001 0.010 < 0.001	
Bakhos, 2019[56]	Esophageal (low)	< 80, 80–89		Chemotherapy Radiation Surgery Multimodality	65.8 57.5 33.3 18.5			36.1 47.8 11.5 2.0	< 0.001 < 0.001 < 0.001 < 0.001	30d mort higher, OS worse
Li, 2020[63]	Pancreas (low)	< 80, ≥ 80		Any treatment Surgery Chemotherapy	78.7 43.0 63.9			44.5 17.9 29.7		Two-year OS worse
Metastatic setting										
Oytun, 2022[37]	Metastatic colon	< 65, ≥ 65		Surgery Adjuvant therapy 1st line CT mono No targeted therapy	44.9 22.3 2.4 29.5		35.3 16.2 10.4 53.2		0.042 0.113 < 0.001 < 0.001	Median OS lower, PFS similar
Liao, 2022[86]	Metastatic Gastric	≤ 70, > 70		Pal. gastrectomy Pal. chemotherapy	39.6 83.1		35.4 48.6		0.439 < 0.001	Median OS worse
Niedersüß- Beke, 2021[82]	Metastatic Colorectal	≤ 70, > 70		Best Supportive care Metastasis resection Resection & systemic	11.2 9.5 21.0		35.7 7.9 9.1		< 0.001 0.339 < 0.001	
Lemelin, 2020[85]	Metastatic Neuro-endocrine Tumors	< 70, ≥ 70		Number treatments Chemotherapy Targeted therapy Radionuclide	3.0 54 30 16		2.0 32 16 5		< 0.0001 < 0.0001 0.0001 < 0.0001	Median OS worse
Part B. Age-related differences in treatment and outcomes for specific treatment-selected patients										
Author, year	Site	Age		Treatment & outcomes	Young (%)	Middle (%)	Older 60 + , 65 + , 70 + , 75 + (%)	Old 80 + (%)	p-value	
SURGERY / TUMOR RESECTION										
Buonomo, 2023[11]	Breast (high)	45–70, ≥ 70		BCS for DCIS No complications	79.0 84.9		86.7 77.7		0.298 0.377	
Luzarraga-Aznar, 2022[22]	Endometrial (high)	< 75, ≥ 75		Adjuvant chemotherapy Adjuvant brachytherapy Adjuvant radiotherapy Complications 5-year PFS 5-year OS 5-year DSS	30.6 31.7 19.0 13.0 80.3 80.1 82.5		16.5 17.5 10.3 20.6 71.2 50.1 74.8		0.014 0.016 0.062 0.120 0.132 < 0.001 0.071	
Matrone, 2020[111]	Thyroid (high)	< 65, ≥ 65		Local treatments Systemic treatment Death rate 5 years	11.2 13.9 9.7		6.4 12.8 13.8		0.17 0.78 0.51	
Inoue, 2019[108]	Hepato-cellular (high)	< 80, ≥ 80		Complications Mortality	24.3 5.8			20.0 8.9	0.5879 0.3373	
Shimada, 2020[107]	Hepato- cellular (high)	< 65, 65–79, ≥ 80		30d mortality 5-year OS Median RFS months	0 62 17		0 65 21	0 62 22	0.86 0.65	
Liu, 2021[109]	Liver (high)	< 65, 65–74, ≥ 75		30-day mortality Morbidity	1.3 39.0	1.0 39.4	1.1 58.5		0.9737 0.0073	
Cheng, 2022[41]	Colorectal (high)	65–79, ≥ 80		Complications			11.9	13.4	0.015	
Kryzauskas, 2021[43]	Colorectal (high)	≤ 75, > 75		Complications 30d mortality	29.7 0.9		37.0 3.1		0.066 0.046	
Cross, 2021[44]	Colorectal (high)	< 80, ≥ 80		Adjuvant therapy colon/rectal Complications 30d mortality	48/56 13/11 0.9/0.4			17/21 23/25 3.3/3.1	0.001 < 0.001 < 0.001	
Sarasqueta, 2019[46]	Colorectal (high)	< 65, 65–80, > 80		Adjuvant CT colon Preop RT rectal	91.9 68.0	76.8 60.4		26.8 42.2	< 0.0005 < 0.0005	
Ogata, 2022[40]	Colorectal (high)	< 60, 60–79, ≥ 80		Post-operative mortality 5-year survival	0 90.8	0 86.6		2.5 52.1	0.072 < 0.001	
Shafiei, 2020[38]	Colon (high)	≤ 69, 70–79, ≥ 80		Adjuvant chemotherapy	82.6		58.5	4.3	< 0.001	
Mazzola, 2023[36]	Colon (high)	< 80, ≥ 80		Adjuvant chemotherapy Overall complications Relapse Survival rate	96.1 23.2 12.6 79.3			16.7 28.6 8.6 67.7	< 0.001 0.525 0.509 0.183	
Liu, 2023[109]	Colon (high)	< 65, ≥ 65		Complications OS 5-years DFS 5-years	24.9 88.0 89.7		37.7 88.6 90.5		< 0.001 0.3 0.38	
Lee, 2022[105]	Gastro-intestinal (high)	< 65, 65–79, ≥ 80		Surgical complications In hospital mortality	39.7 2.4		46.5 2.7	13.9 4.5	0.265 0.674	
Leminski, 2022[89]	Bladder (low)	< 70, ≥ 70		Neoadjuvant chemotherapy Complications (gr3-5) 90d mortality One-year mortality	18.61 25.31 4.47 33.25		17.58 29.09 7.27 46.67		0.772 0.353 0.175 0.003	
Sirithanaphol, 2019[115]	Renal cell (low)	< 65, ≥ 65		Open surgery 90 days mortality Complications 5-year survival	93.2 1.3 12.2 75.4		92.6 3.7 22.2 45.9		0.60 0.46 0.21 0.031	
Joueidi, 2020[17]	Ovarian (low)	< 65, 65–74, ≥ 75		Neoadjuvant chemotherapy < 6 CT cycles DFS CSSS OS	56 10 47.2 81.7 76.2	67 10 33.7 67.3 61.3	62 23 37.2 57.4 56.4		0.028 0.003 0.057 < 0.001 < 0.001	
Pinelli, 2021[18]	Ovarian (low)	< 70, ≥ 70		Neoadjuvant chemotherapy Complete resection Complications Grade 3/4 AE	19 78 9 14		39 87 2 9		0.03 0.32 0.24 0.40	
Laurent, 2022[52]	Esophageal (low)	< 70, ≥ 70		Neo-adjuvant Adjuvant 30d mortality	61.5 41.7 0.5		44.3 23.0 8.2		0.018 0.004	
Suzuki, 2022[55]	Esophageal (low)	< 76, ≥ 76		Neoadjuvant chemo Completion rate NAC Grade 3 AE	77 85 27.5		55 71 42.9		0.001 0.116 0.091	
Baranov, 2022[57]	Esophageal (low)	< 75, ≥ 75		Neoadjuvant chemo Neoadjuvant CRT Open surgery Overall complications 30d mortality	4.5 88.6 18.0 61.5 2.0		2.4 88.6 14.3 67.9 3.3		0.041 0.990 0.058 0.032 0.262	
Kanda, 2019[53]	Esophageal (low)	< 75, ≥ 75		Neoadjuvant therapy Adjuvant therapy Complications	47 12 12		42 2 8		0.562 0.022 0.445	
Klevebro, 2019[58]	Esophageal or gastric (low)	< 75, ≥ 75		Neoadjuvant CT gastric Neoadjuvant CT esophageal Complication esophageal 30d mortality esophageal Complication gastric 90d mortality gastric	60.7 25.0 71.7 2.0 54.9 7.6		8.3 4.8 74.2 6.9 53.3 18.6		0.691 0.421 0.041 0.028	
Esaki, 2019[51]	Gastric (low)	< 70, 70–79, ≥ 80		Additional surgery	70		54.7	20.1	0.001	
Otowa, 2019[48]	Gastric (low)	≤ 69, 70–79, ≥ 80		Adjuvant chemotherapy All complications	76.9 21.8		37.5 39.7	8.9 30.8	< 0.001 0.055	
Komori, 2020[50]	Gastric (low)	< 80, ≥ 80		Surgical complications Postoperative mortality 5-year OS 5-year CSS	29.4 0.5 66.7 78.2			32.4 0 59.6 67.9	0.699 0.99 0.103 0.028	
Perla, 2021[118]	Infra-tentorial (low)	18–64, 65–74, ≥ 75		Any complication 30d mortality	8.07 1.07	15.07 3.18	21.94 6.45		< 0.001 0.019	
Thakur, 2020[117]	Meningioma (low)	< 65, ≥ 65		Radiation Major complication	16.2 8		6.7 8.7		0.053 0.62	
Nia, 2020[121]	Mixed (craniotomy)	< 65, ≥ 65		Major complications Mortality	6.59 1.68		9.23 4.3		< 0.001 < 0.001	
De Leon, 2021[73]	Lung (low)	< 65, ≥ 65		Open surgery Complications	34 42		27 26		< 0.03 < 0.001	
Henry, 2022[65]	Pancreatic (low)	< 75, ≥ 75		Neoadjuvant chemotherapy Adjuvant chemotherapy Major complications 90-day mortality OS months median DFS months median	8 69 28 5 21 16		5 37 31 8 15 12		0.11 < 0.001 0.43 0.18 < 0.001 < 0.001	
Gruppo, 2020[61]	Pancreatic (low)	< 75, ≥ 75		90-d mortality Complications Mean OS months	1.4 35.6 28.5		9.1 45.4 22		0.088 NS 0.909	
Hackner, 2022[62]	Pancreatic (low)	≤ 70, > 70		Adjuvant chemotherapy Major morbidity Mortality OS months DFS months	60 24 2 29.2 14.9		46 33 7 17.1 10.4		0.038 0.167 0.073 < 0.001 0.034	
Malik, 2020[70]	Pancreatic (low)	< 70, ≥ 70		Neoadjuvant chemotherapy Adjuvant chemotherapy OS median DFS median	5.5 61.7 23.4 13.4		3.2 47.9 17.6 12.3		0.524 0.040 0.382 0.525	
Ramanathan, 2019[60]	Pancreatic (low)	< 60, 60–69, 70–79, ≥ 80		Mortality	0.29	0.59	0.58	0	0.7	
Izumo, 2021[68]	Pancreatic (low)	< 80, ≥ 80		Adjuvant chemotherapy In hospital mortality Recurrence rate Median DSS years Median OS years	63 0.2 72 2.8 2.7			39 0 61 2.3 2.2	0.0085 0.74 0.64 0.47 0.46	
LAPAROSCOPIC OR ROBOTIC RESECTION										
Hotton, 2020[21]	Endometrial (high)	< 70, ≥ 70		No adjuvant treatment Complications Death	43 10.5 1.2		32.2 12.9 1.6		NS NS NS	
Peltrini, 2021[24]	Rectal (high)	< 75, ≥ 75		Complications Death during hospitalization	44.3 0.9%		51.9 0%		0.3 0.9	
Xu, 2021[47]	Gastric (low)	< 65, ≥ 65		Undergoing laparoscopic-assisted gastrectomy – complications	7.6		13.4		0.132	
NEO-ADJUVANT THERAPY FOLLOWED BY SURGERY										
Bongiolatti, 2020[123]	NSCLC (low)		< 70, ≥ 70	Complications 3-year OS 2-year DFS	38 61 61.7		46.1 48.5 44		0.47 0.64 0.393	
Liu, 2020[109]	Rectal (high)		< 65, ≥ 65	Complications Neo-adj Complications surgery	12.67 13.70		21.36 8.74		0.099 P = 0.189	
Inanc, 2022[27]	Rectal (high)		< 65, ≥ 65	Complications OS DFS	0.8 79.0 50.5		3.3 68.0 44.7		0.058 0.05 0.311	
Sonal, 2023[25]	Rectal (high)		< 70, ≥ 70	Complications Recurrence rate Mortality rate	11.8 17.9 14.4		22.2 13.1 44.4		0.015 0.284 < 0.001	
Cooper, 2021[54]	Esophageal (low)		< 70, ≥ 70	Treatment completed Complications 1-year mortality	84 49 9.6		78 58 17.3		0.441 0.222 0.2	
Keywani, 2023[49]	Gastric (low)		< 70, 70–74, 75–79, ≥ 80	Not proceed to surgery	77.3	64.0	62.4	68.4	< 0.001	
Oba, 2021[69]	Pancreatic (low)		< 70, 70–74, ≥ 75	Surgical resection Median survival months	80 23.6	50 18.0	35 17.6		< 0.001 0.090	
Sawyer, 2021[67]	Pancreatic (low)		< 70, 70–74, ≥ 75	Surgical resection Side effects	80 19.4		50 14	35 12	< 0.001 0.563	
NEO-ADJUVANT THERAPY FOLLOWED BY RADIOTHERAPY										
Bryant, 2022[116]	Prostate (high)		≤ 59, 60–69, ≥ 70	Recurrence 10-y PCa specific mortality	35.0 8.0	31.8 6.9	26.7 8.53		< 0.001 0.10	
ADJUVANT CHEMOTHERAPY										
Shiraishi, 2023[42]	Colorectal (high)		< 70, ≥ 70	Adjuvant CT stage III Complications surgery	81.8 28.9		51.1 23.4		< 0.001 0.383	
Okamoto, 2022[45]	Colorectal (high)		< 70, ≥ 70	Grade 3/4 AE Dose limiting toxicity	38 87		35 83		0.75 0.52	
Hagerty, 2022[39]	Colon (high)		≤ 45, 50–75, > 75	Adjuvant CT stage III	56	30	7		< 0.001	
Zambrano-Vera, 2021[19]	Ovarian (low)		< 65, ≥ 65	Adjuvant CT Upfront, NACT + CRS/HIPEC Salvage CT	89.3 95.2 66.7		71.4 70.4 36.8		0.14 0.03 0.03	
ADJUVANT THERAPY										
Matsuoka, 2021[124]	Bladder (low)		< 75, ≥ 75	Receiving intravesical BCG after TURBT – 5-year RFS	51.6		59.4		0.72	
RADIOTHERAPY										
Hou, 202[13]	Cervical (high)		< 70, ≥ 70	RT dose ICRT application With chemotherapy 5-years OS 5-years CSS 5-years LRFS	80.4 69.9 89.2 60.4 66.2 82.6		70.3 32.5 52.5 39.1 64.5 82.1		0.002 < 0.001 < 0.001 0.006 0.89 0.91	
IMMUNOTHERAPY										
Nemoto, 2022[91]	Metastatic RCC (low)		≤ 70, > 70	Nivo mono PFS median Nivo mono OS median Nivo and Ipi PFS OS 1-year	5.69 NR 9.70 82.8		7.3 31.1 6.08 86.6		0.607 0.383 0.997 0.714	
Araujo, 2021[84]	Metastatic RCC (low)		< 70, ≥ 70	OS months Time treatment failure	30.8 6.9		25.1 6.9		< 0.01 0.4	
Shiono, 2023[90]	SCLC (low)		< 70, ≥ 70	Number cycles Median PFS months Median OS months	2 4.9 15.9		2 5.5 15.4		0.81 0.18 0.24	
Okishio, 2020[76]	NSCLC (low)		< 75, ≥ 75	Median doses Median PFS months OS median months	5 2.1 14.7		5 2.1 12.3		0.5074 0.5441 0.3272	
Grosjean, 2021[74]	NSCLC (low)		< 70, ≥ 70	Time to failure months Median OS Any significant AE	3.46 11.2 26.6		4.14 11.3 26.6		0.98 0.91 0.99	
Galli, 2019[79]	NSCLC (low)		< 70, 70–79, ≥ 80	Toxicity grade 2 or more Median PFS months Median OS months	35.8 2.8 9.1	32.7 3.5 11.3		37.5 2.6 9.6	0.6493 0.2020 0.5154	
Vithayathil, 2022[92]	Hepatocellular (low)		< 65, ≥ 65	OS months Median DFS Treatment related AE	15.1 7.1 73.3		14.9 5.5 62.1		0.67 0.69 0.11	
Johns, 2021[120]	Mixed (low)		< 70, ≥ 70	Any grade toxicity ≥ Grade 3 toxicity	28.7 14.5		36.1 13.5		0.05 0.71	
CHEMO-IMMUNOTHERAPY										
Storm, 2022[122]	Mixed (low)		< 65, ≥ 65	Chemo-immunotherapy Skin AE (most prevalent)	17.4 45.7		14.4 60.0		0.55 0.036	
Takeda, 2023[78]	SCLC (low)		< 75, ≥ 75	Dose reduction Median PFS months Median OS months	20.4 5.1 14.1		47.4 5.5 12.0			
CHEMO-RADIOTHERAPY										
Zaborowska-Szmit, 2021[75]	NSCLC (low)		≤ 65, > 65	Deterioration PS Complications G3/4 Complete response	31.3 31.3 4.9		15.4 42.3 15.4		0.03 0.15 0.01	
CHEMOTHERAPY										
Kotchetkov, 2023[93]	Lymphoma (low)		< 70, ≥ 70	Full dose Deaths DFS	79.5 17.0 27.3		44 10.6 22.1		< 0.001 0.127 0.401	
Pilleron, 2023[77]	NSCLC (low)		< 75, ≥ 75	Therapy adjusted 2 or more regimens Median survival stage III Median survival stage IV	24 49 15.8 7.7		18 42–44 13.0 7.9			
Potthoff, 2020[83]	Metastatic breast (low)		< 70, ≥ 70	Time progression months OS median months AE (grade 3/4)	6.0 16.4 12.0		6.9 14.5 12.1			
Elias, 2022[64]	Metastatic pancreatic (low)		< 70, 70–79, ≥ 80	First line treatment Second line treatment Third line treatment Median OS months	74.9 43.0 34.4 7.9		70.9 34.5 31.3 6.8	58.4 26.7 31.5 6.2	< 0.0001 < 0.0001 0.3156 < 0.0001	
Koga, 2022[81]	Metastatic pancreatic (low)		< 75, ≥ 75	PFS OS	6.0 11.1		5.5 12.0		0.21 0.43	
Kunkel, 2021[125]	Pancreatic (low)		< 75, ≥ 75	Folfirinox Gemcitabine mono Hematologic toxicity Median survival months	70.8 38.9 56.9 12.7		12.0 72.0 40.0 9.9		< 0.001 0.004 0.144 0.001	
Prager, 2021[80]	Pancreatic (low)		≤ 70, > 70	Median PFS Median OS Adverse Drug Reaction	5.562 10.64 70.4		5.520 10.22 70.8		0.81 0.4	
TARGETED THERAPY										
Marta, 2021[106]	HCC (low)		< 70, ≥ 70	Toxicities skin rash OS Time treatment failure	38.5 8.0 3.0		33.3 9.0 3.0		0.532 0.433 0.936	

TN = triple negative, DCIS = ductal carcinoma in situ, BCS = breast conserving surgery, CT = chemotherapy, surg. = surgical, CSM = cancer specific mortality, pal. = palliative, MPM = Malignant pleural mesothelioma, LCNEC = large cell neuroendocrine carcinoma, RCC = Renal cell carcinoma, STS = Soft tissue sarcoma, OS = Overall Survival, CSS = cancer specific survival, RS = relative survival, mort. = mortality, NAC = neoadjuvant chemotherapy, AE = Adverse Events, CRT = chemoradiation, CT = chemotherapy, preop RT = preoperative radiotherapy, DFS = Disease Free Survival, DFS = Disease Free Survival, NSCLC = non-small cell lung cancer, SCLC = small cell lung cancer, Nivo = Nivolumab, and Ipi = ipilimumab

Regardless of the age cut-off used and the treatment studied, older adults are more likely to receive no treatment or no (neo)adjuvant treatment, and have poorer survival than younger adults. Remarkably, in a study, palliative treatment was administrated in a higher percentage to older patients with anal cancer (16.1%) than younger patients (11.2%) [87], as well as bypass or stoma for rectal cancer; 5%, 10% and 26% for < 65, 65–79 and 80 + years respectively [31]. One study on cervical cancer did not show differences in chemotherapy rates across age categories; 47.3% for under 65 years versus 47.0% for patients of 65 years and older [14]. For the studies that used 80 years as a cut-off [56, 63, 88], age-related differences in treatment were more pronounced, especially in the non-receipt of chemotherapy, with difference going up to 34% [63].

Differences in treatment rates, especially assessing the non-receipt of therapy, were often less than 10% between younger and older patients aged over 60 or 65 diagnosed with a good prognosis cancer, such as breast cancer [12]. An exception to this is one study in patients with cervical cancer, where 62% of the younger patients underwent surgery, compared to 36% in the older group [14]. For patients diagnosed with poor-prognosis cancers such as lung or pancreatic cancer, differences in treatment rates across age groups were most often larger. For instance, in patients with pancreatic cancer, 33% of the younger patients received no treatment versus 67% of the patients over 60 years [59]. For lung cancer, age-related differences were smaller, although a study reported larger differences with 4.4% of the younger patients who did not received treatment versus 20.2% in those over 65, and 54.7% in those over the age of 80 years [71].

### Age-Related Treatment and Outcome Differences for Selected Patients (Table 2, part B)

For patients who underwent surgery, older patients were consistently less likely to receive neo-adjuvant or adjuvant therapy regardless of the cancer type [17, 18, 22, 36, 38, 44, 46, 48, 52, 53, 55, 57, 58, 62, 65, 68, 70, 89].For those receiving systemic treatment, the completion rate was lower in older patients [53, 54, 76]. Overall mortality, 30-day mortality, long-term survival and disease-free survival were worse in older patients in most of the studies. Age-related differences in treatment and outcomes were more pronounced from the age of 80 years, for example in colorectal cancer where more complications and a higher 30-day mortality were recorded: 13% versus 21% for colon cancer and 11% versus 23% for rectal cancer and a 30-day mortality of 0.9 versus 3.3 for colon and 0.4 versus 3.1 for rectal cancer, respectively [44]. Older patients were less likely to receive adjuvant chemotherapy; however, once selected for treatment, their complications and adverse events rates were similar to younger patients [39, 42, 45]. With respect to patients receiving immunotherapy, the number of cycles or median doses received was similar between younger and older patients [76, 90]. Among patients receiving immunotherapy or chemotherapy, overall and progression free survival was comparable across age groups in most of the studies [74, 76, 79–81, 90–93].

Assessing those poor prognosis cancer types, for example pancreatic cancer, complications after surgery are usually not significantly different across age groups [61, 62, 65]. For colon or colorectal cancer, postoperative complications were significantly different between the age groups in some studies [41, 44], while other showed similar rates [36] which might be explained by differences in patients characteristics selected for surgery or not.

## Discussion

The present systematic review describes the recent literature on differences in treatment and outcomes between younger and older patients with cancer. Most articles reported studies on colorectal, pancreatic and lung cancers, and were usually from single centers in Europe or the USA. For unselected patients, older patients still receive less (systemic) treatment and more often no treatment. Besides, survival, regardless the metric used, was poorer in older patients, with more pronounced differences in patients over the age of 80 years. However, for selected patients, especially for surgery, immunotherapy or chemotherapy, complications or adverse events as well as survival were usually comparable between older and younger patients. It is urgent to improve the representation of older adults in cancer treatment research to help narrow down age-related disparities in treatment receipt and outcomes.

It is tempting to speculate why older patients are less likely to receive treatment, and whether this is undertreatment or tailored treatment. Many valid reasons can explain why patients do not receive treatment: patients’ health status and frailty, restricted risk–benefit balance, limited life expectancy, or patients’ preference [71]. However, the lack of scientific evidence on the effectiveness of treatment, specifically in multimorbid and oldest patients [82] can lead physician to rely on their assumptions and stereotypes [94–96].

Older patients are rarely included in randomized clinical trials. Reasons for their exclusion include comorbidities and polypharmacy, making it harder to meet clinical trial eligibility criteria; or the lack of physician knowledge about available trials or concerns about the potential risks and benefits of participation. Furthermore, beneficial treatment effects may be smaller in older patients with cancer due to specific aspects unique to this age group of patients, such as competing deaths, shorter follow-up, or compromised treatment tolerance [97]. Consequently, cancer treatment decisions for older adults are largely based on limited, post-hoc subgroup analyses and extrapolation of results from studies of younger patients [98]. While modified treatment strategies are frequently used for older patients, the evidence for such approaches is poor. Consequently, older adults are at higher risk of under and over treatment, which can affect their quality of life and survival [99]. Preferably, treatment decisions should consider physiological age, estimated life expectancy, risks, benefits, treatment tolerance, patient preferences, and potential treatment barriers [100]. There is growing evidence showing the effectiveness of comprehensive geriatric assessment together with targeted intervention to improve quality of life, reduce the risk of toxicity and complications. This is also recommended by the American Society of Clinical Oncology and the International Society for Geriatric Oncology [101, 102].There is a crucial need for a better representation of older adults in cancer research and observational studies are an important data sources to fill the gap in evidence.

Consistently, older patients selected for treatment have similar outcomes than younger patients, suggesting the appropriate selection of patients for treatment. However, this is not conclusive with respect to the patients who were not selected for treatment but could have benefited from it. It is likely that those older adults who received treatment were fitter than those who did not. Information on frailty status would be valuable to better describe older patients who received treatment compared to those who did not. However, such information is rarely available routinely in observational studies [103]. Fortunately, some tools are being developed to assess frailty in secondary health data [104].

Most studies compared overall survival between younger and older adults; however, older adults have a higher baseline mortality than younger adults. While overall survival in patients with cancer can provide valuable information over short periods, it may not be the most relevant metric for long-term comparisons across age groups as it does not make the distinction between the cause of death. Cancer-specific survival in hospital settings, where the cause of death can be accurately determined, or net survival metrics in population-based studies, where the cause of death may be less reliable, could be better options. These metrics remove the effect of population mortality, making them more suitable for comparing survival between older and younger age groups.

The observational studies included are descriptive studies, and not designed to compare treatment effectiveness. Evaluating treatment effectiveness using observational is complicated by the inherent biases of observational studies such as selection or confounding which can affect interpretation. Such studies require special attention when planning the study, analyzing data and interpreting results; well-thought observational studies in older patients with cancer are urgently needed to fill the knowledge gaps.

Limitations of the present review are the strict selection criteria and the reduction in the items that were extracted from the articles. Moreover, the different definitions and cut-offs used in the articles complicated the overview (both age categories and the outcomes). Most of the selected articles were from European or USA data, highlighting the importance of much needed research from other parts of the world. Besides, most of the studies were performed in a single center; therefore informative larger population-based studies are warranted to reduce the risk of selection bias. Last, for the aim of this overview, we had to select items to extract from the articles, thereby acknowledging that some important items and outcomes such as quality of life or functional outcomes are missing in this systematic review.

## Conclusion

Older patients continue to receive less treatment and have poorer survival compared to their younger counterparts, with more pronounced differences in patients over the age of 80. However, older patients selected for specific treatments experienced comparable complications and adverse events rates as well as similar survival. Given the lack of evidence to tailor treatment for older patients with cancer, it is crucial to increase their representation in clinical trials; this is particularly important for evaluating different treatment options in the oldest and frailest patients.

## Key References

- Drapalik LM, Estes A, Sarode AL, Cao L, Shenk RR, Jarrett CM, et al. Age disparities in triple-negative breast cancer treatment and outcomes: An NCDB analysis. Surgery. 2022;172(3):821–30.
  - This study with the National Cancer Database compares treatment of younger and older patients with triple-negative breast cancer.
- Jauhari Y, Gannon MR, Tsang C, Horgan K, Dodwell D, Clements K, et al. Surgery and adjuvant radiotherapy for unilateral ductal carcinoma in situ (DCIS) in women aged over 70 years: A population based cohort study. Eur J Surg Oncol. 2019;45(8):1378–87.
  - This study evaluates the management of DCIS in women aged 70 or more compared to 50-69 in England and Wales.
- Xie S, Pan S, Zou S, Zhu H, Zhu X. Characteristics and Treatments of Patients Aged 65 Years or Over with Cervical Cancer. Clin Interv Aging. 2020;15:841–51.
  - This study report the differences in characteristics of patients diagnosed with cervical cancer aged over 65 years and under 65 selected from the SEER database.
- Neumeyer S, Tanaka LF, Liang LA, Klug SJ. Epidemiology of cervical cancer in elderly women: Analysis of incidence, treatment, and survival using German registry data. Cancer Med. 2023;12:17284–95.
  - This study provides an overview of treatment and survival differences between younger and older women with cervical cancer using the German Centre of Cancer Registry database.
- De Nes LCF, Heil TC, Verhoeven RHA, Lemmens V, Rutten HJ, De Wilt JHW, et al. Impact of Age on Multimodality Treatment and Survival in Locally Advanced Rectal Cancer Patients. Cancers Basel [Internet]. 2022;14(11). Available from: https://mdpi-res.com/d_attachment/cancers/cancers-14-02741/article_deploy/cancers-14-02741-v3.pdf?version=1654140141
  - This study analyses whether age below and above 70 years is associated with differences in treatment and outcome for patients with locally advanced rectal cancer.
- Birch RJ, Taylor JC, Downing A, Spencer K, Finan PJ, Audisio RA, et al. Rectal cancer in old age -is it appropriately managed? Evidence from population-based analysis of routine data across the English national health service. Eur J Surg Oncol. 2019;45(7):1196–204.
  - This study from England examines the use of radical rectal cancer treatments and associated outcomes in relation to age.
- Hayes L, Forrest L, Adams J, Hidajat M, Ben-Shlomo Y, White M, et al. Age-related inequalities in colon cancer treatment persist over time: a population-based analysis. J Epidemiol Community Health. 2019;73(1):34–41.
  - This study from the UK population-based Northern and Yorkshire Cancer Registry examined if treatment for colon cancer was related to age.
- Keywani K, Borgstein ABJ, Eshuis WJ, Pape M, Versteeg KS, Derks S, et al. Neoadjuvant chemotherapy in older patients with gastric cancer undergoing surgery: a population-based cohort study. Gastric Cancer. 2023;26(5):763–74.
  - This population-based cohort study compared the treatment and survival outcomes of patients under and above 75 years with gastric cancer.
- Bakhos CT, Salami AC, Kaiser LR, Petrov RV, Abbas AE. Outcomes of octogenarians with esophageal cancer: an analysis of the National Cancer Database. Esophagus. 2019;32(10):1–8.
  - This study uses the National Cancer Database to compare the treatment and outcomes of esophageal cancer between octogenarians and non-octogenarians.
- van Dongen JC, van der Geest LGM, de Meijer VE, van Santvoort HC, de Vos-Geelen J, Besselink MG, et al. Age and prognosis in patients with pancreatic cancer: a population-based study. Acta Oncol. 2022;61(3):286–93.
  - This study from the national Netherlands Cancer Registry compares clinicopathological, treatment and overall survival of pancreatic ductal adenocarcinoma of patients aged <60 years to older patients.
- Walter J, Tufman A, Holle R, Schwarzkopf L. “Age matters”-German claims data indicate disparities in lung cancer care between elderly and young patients. PLoS One. 2019;14(6):e0217434.
  - This study studied claims from 13283 German patients with lung cancer and compared therapy between patients aged under 65, 65-74, 75-84 and over 85 years.
- Pham J, Conron M, Wright G, Mitchell P, Ball D, Philip J, et al. Excess mortality and undertreatment in elderly lung cancer patients: treatment nihilism in the modern era? ERJ Open Res [Internet]. 2021 [cited 4AD Jan 1];7(2). Available from: https://openres.ersjournals.com/content/erjor/7/2/00393-2020.full.pdf
  - This study compares treatment and survival between patients with lung cancer in the ages <60, 60-69, 70-79 and 80 years and over.
- Pilleron S, Morris EJA, Dodwell D, Franks KN. Patterns of chemotherapy use and outcomes in advanced non-small cell lung cancer by age in England: A retrospective analysis of the population-based Systemic Anti-Cancer Treatment (SACT) dataset. J Geriatr Oncol. 2023;14(7):101581.
  - This population-based study describes patterns of treatment and outcomes for patients diagnosed with non-small cell lung cancer aged <75 and 75 or over.
- Miller ED, Nalin AP, Pardo DAD, Arnett AL, Huang E, Gasior AC, et al. Disparate Use of Chemoradiation in Elderly Patients with Localized Anal Cancer. JNCCN J Natl Compr Cancer Netw. 2022;20(6):644–52.
  - This study with the National Cancer Database compares patterns of care for treatment of squamous cell carcinoma of the anus in elderly versus nonelderly patients.
- Bryant AK, Nelson TJ, McKay RR, Kader AK, Parsons JK, Einck JP, et al. Impact of age on treatment response in men with prostate cancer treated with radiotherapy. BJUI Compass. 2022;3(3):243–50.
  - This large study using the nationwide Veterans Affairs data analyses the effect of age on treatment and outcomes of localized prostate cancer.
- Gingrich AA, Bateni SB, Monjazeb AM, Thorpe SW, Kirane AR, Bold RJ, et al. Extremity soft tissue sarcoma in the elderly: Are we overtreating or undertreating this potentially vulnerable patient population? J Surg Oncol. 2019;119(8):1087–98.
  - This study, using the National Cancer Database, analyses and compares the treatment and outcomes between young and older patients with nonmetastatic extremity soft tissue sarcoma.

## Data Availability

No datasets were generated or analysed during the current study.
